# Challenges in the delivery of health services for people living with HIV in Dar es Salaam, Tanzania: a qualitative descriptive study among healthcare providers

**DOI:** 10.3389/frhs.2024.1336809

**Published:** 2024-02-28

**Authors:** Masunga K. Iseselo, Joel S. Ambikile, Gift G. Lukumay, Idda H. Mosha

**Affiliations:** ^1^ Department of Clinical Nursing, School of Nursing, Muhimbili University of Health and Allied Sciences, Dar es Salaam, Tanzania; ^2^ Department of Community Health Nursing, School of Nursing, Muhimbili University of Health and Allied Sciences, Dar es Salaam, Tanzania; ^3^ Department of Behavioural Sciences, School of Public Health and Social Sciences, Muhimbili University of Health and Allied Sciences, Dar es Salaam, Tanzania

**Keywords:** HIV/AIDS, healthcare services, challenges, CTC, healthcare providers, Tanzania

## Abstract

**Background:**

Healthcare providers play an important role in the provision of health services in care and treatment clinics (CTCs), as they help clients cope with their HIV/AIDS diseases by providing health education and counseling. Little is known about the challenges that healthcare providers face when providing such services to people living with HIV (PLWH) in low-resource settings. This study aimed to explore and understand the challenges that healthcare providers face in delivering care to PLWH in Dar es Salaam, Tanzania.

**Materials and methods:**

We conducted a qualitative descriptive study to explore and understand the challenges that healthcare providers face when providing health services to PLWH. This study was carried out in the CTCs in the Ubungo District, Dar es Salaam, Tanzania. A total of 10 healthcare providers were recruited using a purposive sampling technique until information saturation was attained. Face-to-face interviews were conducted to obtain the data. We transcribed the interviews verbatim and analyzed them using reflexive thematic analysis to obtain the themes and subthemes.

**Findings:**

Client-related challenges included difficulty in managing antiretroviral (ARV) drug reactions of clients and their financial, follow-up, and index tracking problems; facility-related challenges included inadequate and limited space for CTC services, lack of integrated HIV services, and shortage of staff, which needed collaborative efforts to overcome; and healthcare provider-related challenges included a lack of up-to-date knowledge and limited access to health information. These challenges limit the provision of quality HIV care to the clients.

**Conclusion:**

This study highlights the important challenges that hinder the quality of HIV services at CTCs. The implementation of appropriate infrastructure to promote the privacy and confidentiality of clients is necessary as it alleviates the burden on the jobs of healthcare providers. The findings also suggest that healthcare providers improvise solutions to meet the needs of the clients in the study setting. Permanent resolution is required to overcome the challenges in CTCs. Further research on both providers and clients should be conducted to explore the challenges in CTCs in other similar settings.

## 1 Introduction

Since the identification of the first human immunodeficiency virus (HIV) case four decades ago, the HIV pandemic has spread worldwide, affecting nearly 39 million people and adding 1.3 million new cases by the end of 2022 (1). The epidemic mostly affects vulnerable populations in low- and middle-income countries (LMICs) and some particular groups in high-income countries (2, 3).

The first cases of HIV and AIDS were reported in Tanzania in 1983; and as of 2023, there were nearly 1.5 million people living with HIV (PLWH) (4). This equates to an estimated 4.4% of adult HIV prevalence (4). Despite a high prevalence, Tanzania has managed to control the HIV epidemic over the past decade (5). Between 2010 and 2018, improved access to antiretroviral therapy (ART) led to a decline in the number of new infections by 13%, and the mortality rate for AIDS-related illnesses was reduced in half (6). Chronic management of HIV/AIDS is one of the priority strategies, which includes providing ART to all HIV-positive people in care and treatment clinics (CTCs) regardless of CD4 count and WHO HIV/AIDS staging. This strategy helps them live longer with an improved quality of life (7–9). The first CTC program in Tanzania began in 2004, and about 6,000 health facilities are registered to deliver HIV care and treatment services, including the provision of ART as of 2018 (10).

Healthcare providers play an important role in the provision of health services in CTCs including the provision of emergency medical services, health education to help people cope with their HIV/AIDS diseases, counseling regarding the importance of adhering to their antiretroviral (ARV) treatment medications, and referral services (11, 12). The high prevalence of HIV and the quick adoption of ART significantly increase the need for health workers (13). Attending HIV-positive patients and relatives in the CTCs creates an individual burden to the overwhelming healthcare system (14, 15). This increases the burden on healthcare providers and their profession. The shortage of human resources in the healthcare system has a significant impact on the efficacy and quality of ART services. One individual must perform the duties of two or three people, including those of highly skilled workers, due to understaffing (13–15).

Organizational structure also relates to the availability of equipment, the management of health workers, and the supply of medications. By lowering the standard of treatment, inadequate physical infrastructure and equipment undermine motivation (14, 16). A literature review has shown that the key structural challenges to the quality of care for PLWH include the cost of transportation, waiting times at the clinics, lack of adherence counseling, and limited availability of support services such as peer support groups (17–19). To our knowledge, little is known about the perspectives and challenges that healthcare providers face when providing services to PLWH in Tanzania.

Our study aimed to explore and better understand the challenges that healthcare providers face in delivering care for PLWH in Dar es Salaam, Tanzania. The goal was to understand the challenges that are unique or most pervasive in Ubungo District and how they address these deficits. Moreover, we aimed to gain insight into how providers interpret the needs of their clients and the structural or clinical challenges they face when serving PLWH in Tanzania.

## 2 Materials and methods

### 2.1 Design

We conducted a qualitative descriptive study to explore the challenges that healthcare providers face when providing care for PLWH clients. To understand the events that occur in a natural setting, we employed a qualitative descriptive design that assumes a naturalistic approach and places emphasis on the straightforward descriptions of experiences and perceptions of participants (20). Such a technique is crucial for providing in-depth justifications for a phenomenon that is not well known (21). Moreover, qualitative descriptive studies aim to comprehend an event from the perspectives of those most impacted by its occurrences and interactions (20). This type of design was considered principally appropriate, since the study aimed to identify the challenges that healthcare providers face when delivering services to PLWH (see Supplementary Material S1: SRQR).

### 2.2 Setting

This study was conducted in Ubungo District, Dar es Salaam, Tanzania. Dar es Salaam has a population of around 7 million and is considered one of the fastest-growing cities in Africa and the largest commercial city in Tanzania (22). A previous study reported that Ubungo District had a total of 37 health facilities that provide CTC and PMTCT services, including 23 dispensaries (18 public, 5 private), 6 health centers (3 public, 3 private), and 4 faith-based health facilities (23). In the current study, we purposely selected eight public health facilities to obtain the study participants. A total of 10 (29.4%) participants were selected from the 34 healthcare providers working in the area.

### 2.3 Participants

Healthcare providers who were working in CTCs and directly involved in providing counseling and other services to PLWH were included in the study. There are two types of healthcare providers included in the current study, namely, nursing and clinician cadres. For the nursing cadre, both assistants and nursing officers (NO) were involved in the study. The assistant nursing officers (ANO) had 3 years of training in nursing at the diploma level and the NO had 4 years of training at the degree level. The clinicians in health centers and dispensaries who participated in the study were clinical officers (CO) and medical officers (MO). The COs had 3 years of training at the diploma level in basic and applied medicine, and the MOs had 5 years of training cadre at the degree level (24). Trackers and pharmacists were excluded from the study due to their indirect involvement with the PLWH.

### 2.4 Sampling and sample size

To obtain study participants, we used purposive sampling, which enabled us to get participants with adequate information and experiences to answer our research questions (25). The first author identified the potential participants and invited them for interviews. The sampling continued until we reached the information saturation on the 10th participant, i.e., when no new information was elicited as far as the research question was concerned.

### 2.5 Data collection tool and procedure

Data were collected through face-to-face interviews with healthcare providers. A semi-structured interview guide (see Supplementary Material S1: Interview guide) was used during the interviews, which took place at the clinics of each provider on the day and time of their convenience. The interview topic guide included themes that were mainly aimed to address the primary research questions for the study. (1) What are the challenges healthcare providers face in providing care to PLWH? (2) In your opinion, how do you meet the psychosocial needs of PLWH given the challenge you face? (3) From the perspectives of these healthcare providers, what are the possible strategies for improving healthcare services for PLWH? The questions were followed by multiple probes that aimed to gain a deeper understanding of the information given by the participants. All the authors, who were also experts in qualitative research, conducted the interviews. The interviewers were unfamiliar with the participants, which made it easy for participants to speak freely. The interviews were in Kiswahili, which is the commonly used language by many people in Tanzania. The interviews were audio-recorded (with the permission of the participants) and took place in their respective health facilities. Demographic information for each participant was collected before starting the interview. Since the room was well-illuminated, the interviewer was able to capture the non-verbal cues from the participants. In addition, the setting was free from external voices since the interview was conducted after all clients had left the premises. The interviewers used all possible interviewing skills (26), which increased cooperation and a sense of acceptance among the participants. The interview continued until no information was obtained by adding new participants. That is, we reached information saturation as per the objectives of the 10th participant (27). Each interview lasted for ∼60 min.

### 2.6 Data management and analysis

Before analysis, the audio-recorded data were transcribed verbatim by a research assistant who was an expert in qualitative research. We cross-checked the transcription process by listening to the original audio and comparing them to the transcripts to ensure the exercise was correctly performed. Some identified typographic errors and omissions were corrected. We analyzed data using reflexive thematic analysis (28) and used additional techniques to guarantee a reliable and credible analysis. First, we used a team-based approach to code the transcripts. In the initial round of coding, all transcripts were coded using an inductive coding strategy to generate an initial codebook. To do this, each author separately read two transcripts that were chosen at random, after which they came up with a list of possible codes. We then discussed the differences, found the connections between the codes, and created a single codebook. We used the updated codebook to improve and evaluate the fit of two more transcripts. As we developed and finalized codes, code definitions, and code structure, this process took place twice more. Deductive codes from the literature were then added to our codebook to better reflect the difficulties healthcare providers encounter when delivering care in CTC (17, 28–30). Once the final codebook and code meanings have been agreed upon, we used NVivo 12 software (31) to apply the final codebook to all transcripts. All interviews were coded by the first and second authors to ensure consistency in the application of the codebook. During the coding process, memoing was used to obtain the first concepts (32). Memos contained the original concepts, ideas, and thoughts of the authors regarding the data. They also served as a space for researchers to reflect and produce audit trails of our analysis and theme development procedures (33, 34). We examined the coded data to obtain patterns and possible themes and specifically looked at the data to comprehend the difficulties that healthcare providers encountered when delivering services to PLWH. Potential themes were investigated, compared to the data, revised, and finally decided upon to represent a central concept. We narrowed our selection of pertinent quotations to include quotes that best exemplified these themes. Our findings were grouped into three primary themes that highlighted the challenges in providing healthcare services to PLWH in the study settings.

### 2.7 Trustworthiness

Using the Stahl and King criteria (35), the rigor and reliability of data were assessed based on their credibility, dependability, conformability, and transferability. The researchers collected and analyzed the data in the field for an extended period to increase credibility. Peer debriefing and constant data comparison were employed to improve dependability. Additionally, codes and categories were shared with two faculty members who were also competent in qualitative research for the external validation of the selected and classified codes. Sampling mode, questions development, the method of coding, and category extraction and change were documented to provide appropriate conformability.

### 2.8 Ethical approval

Ethical approval to conduct this study was obtained from Muhimbili University of Health and Allied Sciences Review and Ethics Committee (MUHAS-REC) with Ref. No. MUHAS-REC-2-2020-093. Further, permission to conduct the study was obtained from the Ubungo Municipality Council. Before the commencement of data collection, all participants were informed about the objectives, expected benefits, risks, and significance of the study. We informed participants about the voluntary nature of the study and their right to withdraw from participation in the study at any time. Privacy and confidentiality of the participants were ensured by collecting data in a private room and de-identifying the personal information from all study documents. Upon agreement of the participants to participate in the study, written consent was obtained from the participants before undertaking data collection.

## 3 Results

### 3.1 Characteristics of study participants

A total of 10 healthcare providers were interviewed in Ubungo Municipality. Of these, three (30%) were males. The mean age of the participants was 42.1 years (SD ± 10.9). Two (20%) were MO and two (20%) were CO. In addition, six (60%) of the participants were nurses, of which five (50%) were ANO and one (10%) was a nursing officer (NO). Their working experience ranged from 4 to 10 years, with a mean of 6.1 years (SD ± 2.2) (Table 1).

**Table 1:** Sociodemographic characteristics of the participants.

Characteristics	Frequency	Percentage (%)
Gender		
Female	7	70
Male	3	30
Age (years)		
25–35	3	30
36–45	4	40
46–55	2	20
56–65	0	0
66–75	1	10
Cadre		
Clinical officers (CO)	2	20
Assistant nursing officers (ANO)	5	50
Nursing officers (NO)	1	10
Medical officer (MO)	2	20
Duration of working in CTCs (years)		
<5	4	40
5–10	6	60
>10	0	0

### 3.2 Themes identified

The themes identified are challenges related to clients, health facilities, and providers. Reactions to ART, follow-up and index tracking, and financial problems are client-related challenges. Limited space, lack of integrated health services, and shortage of staff are health facility-related challenges, whereas lack of up-to-date knowledge is a health provider-related challenge.

### 3.3 Client-related challenge

#### 3.3.1 ARV reactions

Participants reported being bothered by the side effects of ARV drugs on the clients. They expressed that they were talking about the side effects of efavirenz (EFV), which causes problems such as drowsy, particularly at night. They added that every drug has its side effects, ARVs, like any other medicine, cause side effects on prolonged use. To lessen such side effects, they advised their clients to take the ARVs at night as in one of the statements below:

*“Therefore, we only advise them to take it early; i.e. at 8 pm. There are people we advise to take it earlier than that time. In others, after a few weeks, the situation* (ARV reaction) *just ends. They become used to the drug. Others take up to six months, to be used to the drug.”* (Female, ANO)

Other participants mentioned that the clients experienced other side effects that they felt uncomfortable with. They expressed receiving different complaints related to the medication taken on a particular day. Some clients reported having developed skin rashes or their skin started turning yellow. This is exemplified in the statements below given by one of the participants:

“…*in the beginning, before we started TLD* (Tenofovir, Lamivudine and Dolutegravir,) *I met two or three cases, they had developed jaundice and I tried to trace them, I referred them to Muhimbili hospital and they did tests and treatment. Later they became fine.*” (Male, MO)

However, participants expressed that the introduction of a single dose with a combination of three (TLD) drugs has solved the side effects caused by EFV. They said that the new drug combination has no EFV component. They elaborated that the transition from TLE (dose with EFV) to TLD [dose with dolutegravir (DTG)] has been a great advancement in care and treatment clinics for clients with HIV as expressed in the following statement:

“…*those were the efavirenz, now they are not used in this TLD combination, they are not used because we have one that started to be used in 2018, it does not have efavirenz.*” (Female, CO)

#### 3.3.2 Follow-up and index tracking problems

Participants described the challenges they face when their client is diagnosed with HIV for the first time. They said if there was a client not ready to start medication, they would continue to talk with him/her because they had the preliminary information such as phone number and the location. This was easy for them to trace the client because, during enrollment into the CTC, they had to start marking (mapping) to know where the client lives and their close relatives. In addition, they could track the residential address of the client through the community, or if the client was still reachable through the phone, they could track and schedule a time for him/her to return to the clinic. However, they accepted that it was a great challenge as evidenced by the following participant:

“…*there is something we call index tracking. If one has come for testing, we find that it is reactive* (HIV Positive), *we take the phone number, do you have a partner (yes), I have a partner; we take phone numbers. We as service providers are doing the work of tracking with our techniques.*” (Male, MO)

The participants acknowledged that some clients may give a map different from the area where he/she lives. This gave the healthcare providers difficulties in following them up. The participants also added that many clients tended to change residential addresses. They said that the client may be taking ARV in their health facility but can stop and go to test again in another health facility and start taking medication there without prior information as elaborated below:

*“Alternatively, he gave you a different map of the area where he lives, you see, if you follow him to his home, you cannot find him. In addition, many customers tend to change residences. Maybe he has taken ARVs from this facility, but sometimes he stops and goes to test and take ARVs from another health facility.”* (Female, ANO)

The participants also witnessed challenges in tracking sexual partners who were not aware of their own HIV status. They said to get challenges, particularly in convincing them (sexual partners) to come to test for HIV if their partners tested positive. However, they expressed to use of different techniques to motivate the other pair to come to the clinic to check their health status as described below:

*“It is his/her choice. But we continue to talk to them, explaining the benefit of caring about their health. So, such clients exist. In such cases, we meet; one partner has already tested and is on medication, but the other partner is not ready to test. Others may tell you that, I do not want my husband to know or I don’t want my wife to know. We have techniques to convince the partner to come here to the centre to test or if he says we should follow him/her we also do so.”* (Female, CO)

The participants also expressed that some clients faced different challenges that made them forget their clinic date. They added that due to the challenges of life, for example, the client might have traveled, and the other client might have personal responsibilities that found themselves in a stressful situation such that they could not remember the date of the follow-up clinic. Further, the participants expressed that some clients might come on a date different from their schedule and explained that they had to be flexible and accept to attend to their clients appropriately. One participant stated:

*“That the client you have scheduled a date for, the client comes on a date that is not for scheduled him/her. So we always agree with them that this is who he is and that's how he decides to come. So we receive him and we give him instructions that the next day, try to come on your date. For some reason, you find that he has skipped the date to refill the drugs, and he has not even taken the medicine.”* (Male, CO)

Participants also reported sending text messages (SMS) using mobile phones with the content “Jali afya yako” (mind your health) to remind the client of the date of their clinic. They stated that they had instructed their client that when they see such a message, it was a reminder that the clinic date is due so that they can start preparing as stated in the following:

*“Moreover, we send an SMS through the message system, and he sent a message; ‘remember your date, you failed to come to your date”, and if tomorrow we send him a message, ‘you have to come tomorrow, we just send a message; so whoever reads the SMS, can understand it.”* (Male, MO)

Another important challenge that was pointed out by the participants was an increase in viral load when they missed attending the CTC for ARV refill and counseling. They said attending the clinic was important for continued health check-ups and counseling. They emphasized that the clinic was the place where they could vent their life stress and other constraints that might have encountered in the community. They emphasized that if this opportunity was not utilized, the clients might end up drinking alcohol contrary to the medication guidelines. Therefore, missing the clinic caused an increase in viral load due to inadequate medication refills and poor adherence as verbalized by the participant below:

“*The challenges that I find with the clients themselves, especially when they miss coming, will have a high viral load. So it will take me some time now for me to counsel him* (give advice) *until I bring him back to lower the viral load, it is a little difficult. For example, if you see the young man who entered here* [interview room], *I have counselled him not to drink alcohol, but he has come here, he is nzwii* [heavily drunk].” (Female, NO)

#### 3.3.3 Financial problems

Financial problems were one of the challenges expressed by participants. They said that most clients had insufficient income to meet their needs. In addition, they reported that having been diagnosed with HIV increased their financial crisis as more money would be needed for transport to and from the CTC. They stated that the financial problems of clients impacted also the healthcare providers that sometimes they had to give some amount of money to their clients who were unable to meet their needs including prescribed medication as in the statement below:

*“I have had a client who was sick, he went to be treated somewhere, not here, but he was prescribed medicines to buy, it was like two medicines, I don’t know which ones, he came here and felt bad that he couldn’t buy those medicines…some medicines he got from the hospital, but it was difficult to get other medicines. So when he reached here, he brought his card with him. I felt compassion from my heart.”* (Female, ANO)

Another participant who was entirely emotionally touched by clients with financial problems added:

*“Economically, some lack fare to come to the facility until they fail how to attend to get those services, as those other services are also far away and it becomes difficult to reach them. How do you help such a person who has missed the fare?”* (Male, CO)

Other participants expressed that the client had difficulty getting the usual meals apart from a balanced diet, and the lack of income compromised their counseling services. They said, in essence, that clients were counseled on getting nutritious food that could help to boost their body immunity. Taking into consideration the low income and financial problems incurred at the time of HIV diagnoses, the participants reported having caused problems in service provision as it was beyond their control as stated by one of the participants:

*“That of income and nutrition is beyond our control. Because you can tell him that these drugs are boring. It is necessary to eat a lot and get a diet, that is, we say a balanced diet, and we say it is a complete diet. But he doesn’t have the ability even though we only direct him to use the things he has in his environment. But economically, speaking now, maybe I can say that I can provide whatever I cannot, so it is a challenge that may be out of our power.”* (Female, CO)

The participants also augmented that the financial problems of clients also impaired their attendance at the clinic. They said that to enable them to attend the clinic or return to their homes, they often used their own money so that clients would be able to travel back to their home or have some meals for the day. This was given by one of the participants below:

*“…Sometimes you give someone the fare, and you take it out of your pocket, and someone else tells you, I could not come because I do not have the fare, or I do not have the fare to come back. In the end, you will give him the fare, you say, I am helping you, take this one thousand or two thousand come tomorrow or the day after tomorrow.”* (Female, ANO)

### 3.4 Health facility-related challenge

#### 3.4.1 Limited space for the provision of health services

The participants expressed that the main challenge was the space to provide services at the CTC. They said the client needed privacy and did not like intermingling with other clients in health facilities to maintain their confidentiality. They said lack of privacy and confidentiality was the likely factor contributing to the attrition of clients to other health clinics. This was because some clients found it difficult to share their issues with the healthcare providers. However, due to the limited space, sharing confidential information was difficult in the presence of three or four healthcare providers in the same room.

*“Our CTC is like what you see; it doesn’t have a nice waiting room like the ideal one. We do not have the privacy to serve clients, someone can come until he sees that the number of other patients has decreased and then he can enter. If there is no privacy here while serving our clients, then it is a problem.”* (Female, ANO)

The participants described that there are several solutions to the problem of limited space for CTC services; they said that they had to schedule their clients to come either early in the morning or late in the afternoon when other clients had left the health facility. They elaborated as an important strategy to make the services go on instead of waiting for the construction of a new building, which takes a long process due to lack of funds. Sometimes they said that they had to shift from one room to another with the client so that they could provide a service with satisfactory privacy as commented below:

*“We can move with him, we came with him, as you can see here we can come here and talk to him. Then those who know themselves, often we like to tell them early in the morning, before people come, if it is to take medicine, they come in that morning at two o’clock. And others decide that I should come during the day, not knowing that there is no privacy here. So I ask you to come in the afternoon at one o’clock or two o’clock.”* (Female, ANO)

Some participants stated to have a single, multipurpose room that encompasses all activities that take place in the clinic on a particular day. Also, in one of the health facilities, it was expressed that they used to conduct CTC services in a container that had the same room for every needed service. They verbalized that the same room was used for pharmacy, data storage, counseling, and an ARV dispensing as stated by one of the participants below:

*“As I said, the space in the CTC room is the same, only what we offer. Here is the data, everything is there, your files are there, and medicine is there. Even counselling is conducted there, there is very little space.”* (Female, NO)

#### 3.4.2 Lack of integrated HIV health services

The participants also expressed that clients with multiple problems that needed various solutions were referred to other health facilities for further medical management. They said that referring to other health facilities even with minor issues that could be managed at the clinic was a challenge to them. The lack of medication in their clinic was mentioned as an example. They stated that some of the health facilities occasionally ran short of ARVs. In such a situation, they used to write prescription forms and send the patient there so that the client received the needed services:

*“A centre like this one has never run out of medication. You write a prescription form and you send the patient there, even though it seems like a hassle, but what should I do now? But we always try to make sure that we get the medicines.”* (Female, NO)

The participants also pointed out that the lack of expertise and medications for opportunistic infection in their health facilities was a stumbling block for smooth continuity of care. They mentioned that if there was a complication that needed a different management approach, they took the client to those centers that had the availability of drugs. They also added that for those who had developed jaundice, they tried to do a liver function test in other health facilities. Because they had limited tests in their centers, they had to refer the clients as expressed below:

*“If perhaps the client has co-morbidities like opportunistic infection such as skin disease or maybe he came with STIs and things like that, we direct him to another clinic where he can get different services related to ART.”* (Male, MO)

The participants unanimously expressed the need for drugs for the treatment of opportunistic infections in their centers. They said that the presence of such medications could help to reduce the disturbances of clients to refer to other clinics for medication only. However, on several occasions, they faced a shortage of such drugs forcing them to refer the client to other health facilities. In that case, they additionally stated the importance of integrated health services where the clients should get all the needed services in one health facility. This could improve the quality of care for the PLWH as described below:

*“I wish that when clients come to CTC, their package ends at CTC; that is, they should get all services at our CTC so that we can reduce the client's movement. For example, there should be enough medicines (ARVs) and we should have access to tests and things like that.”* (Male, CO)

The lack of specialists in the clinic was also mentioned as the cause of the lack of integrated care services at the CTCs. They described that clients come with different non-communicable diseases such as cancer, cardiovascular problems, and mental illness. Such conditions need specialists who can be consulted without making the client move from one center to another. They added that it was also important to reduce cost and time management for the clients so that they could do other productive jobs for their income. One participant said:

*“One client may have two or three diseases; for example, mental illness, cancer or high blood pressure that needed to be treated. That is, those who need these services to be attended in one place. That is the expertise was there to manage patients like this because could be better off rather than sending the client to another unit that deals with the problem that is not present here.”* (Female, ANO)

#### 3.4.3 Shortage of staff

The participants expressed concern that they were very few to the extent that they were overworking beyond their capacity. They said that the HIV CTC was the place where they talked with the client to evaluate the progress of the treatment and the overall condition in general. In addition, CTC services have multiple tasks that need to be fulfilled before the patient leaves the health facility. These were challenging activities for a single person to attend more than 15 PLWH at the clinic as described by the participant:

*“The problem is that the staff is very few and in reality, HIV CTC is a very special unit, not just to attend to the patient and leave, but also offers other health services crucial for client welfare. For example, I provide service for more than 15 people, how much do I talk to 15 clients with this shortage? You find that you are going to give education to pregnant women again, you are coming again for HIV counselling and you have not yet given DBS* (dry blood spot), *you have not given your viral load.”* (Female, ANO)

The participants also added that they were doing multiple jobs due to the lack of staff. They expressed that three types of staff are supposed to be in the HIV CTC, namely, a doctor, a nurse, and a tracker. They described that each staff member had been assigned a specific task to fulfill in the clinic. However, due to the shortage of staff, they found that one person can perform multiple tasks when one or two of the staff are absent:

*“There is a period when you are alone, so you cannot divide yourself into different parts. For example, there is another unit, other activities are going on within the unit, apart from seeing patients, there are testing patients and other activities like that, so sometimes you have to do all the tasks.”* (Female, CO)

The participants described that due to multiple works performed at the HIV CTC, sometimes they become tired. They added that they would attend to more than 30 clients including non-HIV-positive clients who needed other services. In that case, they had to extend working hours to 5 p.m. to attend to all clients on a particular day as expressed below:

*“I had more than 30 clients so you find that you are working and you are working until 5 p.m. For past clients, I have released a viral load at 3 p.m., that is, sometimes you are overworked. That is the only challenge that I see here.”* (Female, NO)

On the other hand, the shortage of staff was perceived positively by some participants. This was described as a learning process that increased their knowledge and skills in a wider perspective of CTC activities. They verbalized that having been left alone or doing a lot of activities helped to gain experience that could enable them to work as doctors, nurses, pharmacists, or laboratory technicians. They had to learn all the work related to HIV CTCs as described below:

*“But here you do everything and many small stations are like that but it helps me. I get the experience of all things. That is, I can do it anywhere there is no doctor, no pharmacy, no lab technician; I can do it. Because in the beginning, it was counselling only, I did not even give medicine, I didn’t know the names of many things, I didn’t know what it was, it was a challenge at that time.”* (Male, MO)

Sometimes shortage of staff was described as a loss to follow-up as some clients left the CTC without getting the needed services. They said clients were not comfortable when they came and found other clients waiting for service. Most clients did not like to queue or stay for a long time for fear of disclosure of their HIV status. The participants further described that, due to a shortage of staff, a single healthcare provider can start with other services such as reproductive and child health which might cause HIV CTC services to be delayed. This caused the clients to start complaining because of delaying getting the services and hence decide to leave without being attended to. This makes to lose a client in their health facilities as commented by one of the participants in the following statement:

*“If he can come and find us there, we have many patients, we are not in the clinic, we are not in the CTC, so if he comes there and stays for a long time, you will find that he has started to complain, or maybe you will find him sitting and seeing that you have not taken care of him in time, he may leave without getting services.”* (Male, MO)

### 3.5 Providers-related challenges

#### 3.5.1 Lack of up-to-date knowledge

The participants expressed that up-to-date knowledge is important for the provision of quality care to clients. Some participants stated to have attended a 2-week training on PMTCT. Despite the training, they said that there was no difference between HIV CTC and PMTCT services. However, they merely described the difference in the aspect of prevention of HIV infection among pregnant mothers. They described that such training was obtained many years back:

*“I used to get it but now it was just like refresher training, it was for two weeks and then for a long time ago, that is, it can reach up to six years. I don’t know maybe it was in 2015 or 2013…2016; I don’t remember but it has been a while.”* (Female, ANO)

Some participants further lamented the lack of training and wished to get on ART refresher courses, to remind themselves of the challenges that the clients face, particularly those facing other sexually transmitted infections (STIs) such as gonorrhea and syphilis. They also emphasized the need for training related to ARV and other medications. Knowledge of drugs particularly of drug pharmacokinetics, pharmacodynamics, and drug reactions was as important as any other training for the benefit of the clients and the quality of care provision in general. This was expressed by one participant in the statement below:

*“You know HIV CTC is not a joke job. You can inject someone with a powercef* (Ceftriaxone) *and will go off* (he will die). *HIV CTC is an absolute blessing. That is, I can make the client; you the service provider stop the medicine, or take the medicine properly, or come to the clinic or not come.”* (Male, MO)

The participants asserted that they needed training related to the new ART treatment regime (TLD). They said the knowledge of the new ART treatment regime is highly needed for all health care providers. This is because most clients aspired to get it and leave behind the former regimen (TLE), which caused side effects due to the presence of EFV. They stressed the importance of such training basically for the improvement in the management of drug reactions and what to expect after the administration of the drugs. This was also important for information provision to the clients as it was part and parcel of the HIV CTC health service packages. Therefore, knowledge of new drugs was also a challenge. This was emphasized by one of the participants below:

*“But there is a new regime that came out for giving medicine and there are these medicines that have come, they are new, there is also a way to give them medicine, I don’t know three months, I don’t know six months, you are looking at it due to clinical science and the illegibility they are saying, I haven’t found them yet. I only do the on-the-job training that I am instructed by my colleagues.”* (Female, CO)

#### 3.5.2 Limited access to health information

The participants also needed access to the HIV Care Guidelines which were developed by the Ministry of Health to guide healthcare providers in CTCs and other areas. However, they lamented that the guidelines have not been distributed to the health facilities so that they can be used by the care providers. Although the Ministry of Health uploaded most of the guidelines on the websites, the participants described that not every health facility staff can access the website to download and use the document. This was described as a major challenge and the healthcare providers were not updated on the new knowledge of the provision of care in the CTC as evidenced below:

*“The guidelines are a bit of a challenge because right now I see that they are not being brought anymore because they have been uploaded into the website. However, it is not that every office has access to the networks, the devices like computers may be there, but where do you get the internet? In other words, the guidelines are posted on the ministry's website to such an extent that even the new knowledge is hard to find.”* (Male, CO)

## 4 Discussion

Our study aimed to explore and better understand the challenges faced by healthcare providers in delivering care for PLWH in Dar es Salaam and how they address these deficits during care provision. Client, health facility, and healthcare provider challenges were elicited from this study. Healthcare providers used individual innovation to face such challenges when providing health services to the study population.

The most prominent client-related challenges as revealed in the current study were ARV drug reactions. As in any other medication treatment for other chronic diseases, the experience of drug reaction is a common phenomenon and particularly higher occurrences are seen at the beginning of ART (36–38), which may decrease in intensity of continued use of the drug. The role of the healthcare provider is to encourage the client to use the drug at the time, which gives a minimal side effect as reported by study participants in our study. The reported skin reaction and dizziness were caused by EFV, which is one of the components of first-line ART and administered once daily. Since adherence to ART is the key determinant of virological suppression in PLWH, the introduction of DTG-based regimens in Tanzania (10) with fewer side effects has improved ART adherence (39, 40). However, a tenofovir, lamivudine, and dolutegravir (TLD)-based regimen is not adequately available as in the study area. The reason may be that the drug has not been extensively distributed in the lower levels of health facilities including dispensaries. The government should make an effort to ensure that this combination is available for the benefit of the PLWH.

The follow-up and index tracking problem as voiced by the study participants may be due to the emotional distress of the client once they test HIV-positive, leading to providing a fake location and changing their mobile phone number. The problem may also be caused by self-referring to other CTCs. Similar losses to follow-up and self-referral have been reported in other studies (41–43). This indicates that there is a lack of skills among healthcare providers in handling clients newly tested for HIV-positive in CTCs. A study in South Africa reported several challenges relating to the roles and responsibilities of the different healthcare providers in the tracing cascade of clients who tested HIV-positive (43). Proper patient documentation with actual residence address is a crucial aspect of adherence. Furthermore, substantial investment in healthcare staff training on the proper linking to health facilities and communities is needed to improve routine tracing.

Tracing the index sexual partner for testing was also a challenge healthcare providers faced as part of controlling the spread of HIV. This may be described by the fact that HIV status disclosure rates to sexual partners are low in Tanzania, despite the benefits it confers to both partners (44, 45). This reluctance to self-disclosure to their sexual partners posed challenges to our study participants in tracking their partners for HIV testing. Although clients were given documented dates for their follow-up clinic, some of them forgot to come to the clinic. The reason for this can be due to life stress and other social responsibility. It is the responsibility of an individual to take the initiative to attend the prescribed dates for the clinic. However, healthcare providers use different mechanisms including text messages (SMS) to ensure that clients are reminded to attend the clinic as scheduled. An automated text message is a feasible and effective way to increase follow-up in HIV-tested individuals in resource-limited countries (46). The use of text messages is a good practice that should be promoted to improve follow-up for HIV-positive clients in other health settings in Tanzania.

The reported increase in viral load among HIV clients when missed ARV refill and counseling emphasizes the importance of adherence to medication that is proven to reduce the viral load (47–49). In addition to ARV provision, the study participants in the current study have shown great effort to trace their clients for HIV counseling as an important aspect of care packages that increase the self-esteem and positive living of clients. Likewise, other studies in Tanzania have reported similar practices that improved the standard of living for PLWH (11, 12).

The financial problems of clients due to reduced productivity of the individual and family members impacted the provision of care in the study population. One of the roles of healthcare providers is counseling on a balanced diet and other healthy lifestyles (50). However, it is a challenging situation to counsel HIV clients who can hardly get a single meal for the day. Moncyk et al. (51) recommended that integrating a family perspective into HIV nutrition interventions and programs in Tanzania has the potential to influence positive outcomes and slow disease progression during the financial crisis. The findings in this study suggest that healthcare providers should strive to counsel HIV clients to choose cheap and readily available food materials.

Concerning the health facility-related challenges, limited space for the provision of health services for people with HIV can be attributed to inadequate funds from the government to construct infrastructure for CTCs. Poor infrastructure compromised privacy and confidentiality among people living with HIV, thus reducing clients attending the clinic for follow-up and ARV refills (52). Ssenyonjo et al. (53) suggested that the governments in low-income countries need to take action accompanied by measures to address bottlenecks in the public financial management system including the budget for increasing infrastructure for HIV CTC services. This could improve the healthcare provision as reported by our study participants.

The lack of integrated HIV health services in the CTC as reported in our study is the main challenge for the provision of quality care. Many factors can contribute to this problem including a lack of specialists for a particular condition and an inadequate supply of other medications for the treatment of opportunistic infection (11, 54, 55). Our study stressed the importance of the integration of health services into HIV care as a valuable strategy to boost the sustainability of the HIV response. A systematic review of literature and meta-analysis revealed the integration of HIV services and other health services tends to improve health and health systems outcomes (56). Integrated management of chronic diseases is a feasible strategy for the control of HIV. More effort should be directed to the redistribution of health professionals and specialists to the lower CTC. This can facilitate the provision of quality care in these health facilities.

Shortage of staff is another health facility-related challenge reported in this study. This can be described by the limited employment opportunity for the health sector with the non-replacement of retiring personnel. A study in the Mbeya region in Tanzania reported that the shortage of staff at the CTC can be attributed to the lack of motivation (15). As reported in our study, this shortage of staff affects the quality and effectiveness of ART provision among the study population. To minimize the effect of this shortage, the study participants have to adjust by extending work hours and performing multiple tasks to provide the care needed to their clients. Similar findings have been reported in other studies whereby one person had to do the tasks of two or three people (14, 15).

Although multitasking was viewed positively by our study sample, the quality of the service outcome was impaired due to exhaustion and limited resting time. This also causes increased waiting time for clients which has a negative impact on their clinic attendance. This is an interesting area that needs further exploration in the future.

As a provider-related challenge, lack of up-to-date knowledge is an important bottleneck for HIV quality healthcare services. The current study has identified several knowledge gaps in the management of clients with HIV in CTCs that limit the ability of healthcare workers to diagnose and manage other conditions in the context of HIV care. As recommended by Leung et al. (57), improving the education of healthcare workers is essential for quality HIV care in the study setting. As communicated in the study, healthcare providers have limited access to health information, particularly to the guidelines related to the care and management of HIV clients. The government needs to allow access to the health materials and guidelines for this lower level of health care provisions. This can help update their knowledge thus smoothening the delivery of health care services to PLWH in their respective clinics.

This study has limitations that should be considered when evaluating the findings. Only the perspectives and opinions of providers on the difficulties they faced in providing HIV health services were included in this study. The perspectives of clients were not evaluated. Self-reported experiences of participants may be biased by social desirability and recall biases. The participants could also be influenced by the anxiety of the face-to-face interview and by the status of the interviewer (from a Medical University). Although the generalizability of the qualitative findings is not an expected attribute outside the study setting (58), this study does raise several important issues related to the challenges of healthcare providers in the delivery of HIV health services in CTCs that are worth taking into account in improving care for clients with HIV in Tanzania.

## 5 Conclusion

The findings of this study highlight some issues that hinder the quality of HIV CTC services and should challenge the health authority in the study area to improve the situation. Inadequate and limited space for provision of care is the main drawback that needs to be addressed. These include establishing appropriate infrastructure to protect the confidentiality and privacy of counseling, which lessens the stress on the jobs of healthcare providers. The findings suggest that healthcare providers improvise solutions to meet the needs of the clients in their workplaces. However, a permanent resolution is required to overcome the challenges in CTCs including integrated health services for PLWH and availability of ARVs with fewer side effects. Further research that will include both providers and clients should be carried out to have an overview of the situation in the whole country.

## Data Availability

The original contributions presented in the study are included in the article/**Supplementary Material**; further inquiries can be directed to the corresponding author.
