# Supplementary material for: Challenges in the delivery of health services for people living with HIV in Dar es Salaam, Tanzania: a qualitative descriptive study among healthcare providers
Source: Front Health Serv. 2024 Feb 28;4:1336809. doi: 10.3389/frhs.2024.1336809 (PMC10933121; doi:10.3389/frhs.2024.1336809)
Supplement: Supplementary file 1 [file Datasheet1.docx]

**Supplementary material 1: Interview guide:**

**Explore healthcare providers' perceptions and challenges in meeting the treatment and psychosocial needs for PLHIV.**

1. For how long have you been working with PLWHIV?
2. How do you finds working or providing health care services to PLWHIV? (Probe whether is it easy or difficult and why)
3. How do you see your ability in providing care to PLWH (knowledge and skills in providing care to PLWHIV? (probe whether trained or not or what makes it possible for them to be able to provide such care/services)
4. What challenges do you face in meeting the treatment needs for PLWHIV? (probe personal and facility-related challenges, strategies do you take to deal with those challenges)
5. In your opinion how do you meeting the psychosocial needs of PLWHIV as you provide services to them?
6. Is there anything else regarding this topic that you want to share with me before ending this interview?
